# Preparing for Vascular Surgery Board Certification: A Comparative Study Using Large Language Models

**DOI:** 10.7759/cureus.83848

**Published:** 2025-05-10

**Authors:** Sonal Kumar, George Y Tadros, Taylor E Collignon, Otto Montero, Sophia Bampoh, Morris Sasson, Alberto Lopez

**Affiliations:** 1 Vascular Surgery, Ross University School of Medicine, Miramar, USA; 2 General Surgery, Cleveland Clinic Florida, Weston, USA; 3 Vascular Medicine, Lake Erie College of Osteopathic Medicine, Miami, USA; 4 Vascular Surgery, Cleveland Clinic Florida, Weston, USA; 5 Vascular Medicine, Cleveland Clinic Florida, Weston, USA

**Keywords:** ai performance evaluation, american board of surgery, artificial intelligence, artificial intelligence in surgery, board exams, comparative study, large language models, vascular education and self-assessment program, vascular surgery, vascular surgery education

## Abstract

Introduction and aim

Large language models (LLMs) are transforming medical education by offering innovative methods to enhance teaching and learning. Despite their demonstrated potential, research on its use in vascular surgery is limited. This study aimed to evaluate and compare the effectiveness of LLM in preparing for vascular surgery board certification exams, exploring their potential as educational supplements.

Methods

We selected 269 text-only multiple-choice questions of 642 from the Vascular Education and Self-Assessment Program (VESAP) version 6. We excluded 143 image-based questions. One independent reviewer input questions into the following four AI tools: ChatGPT 3.5 (San Francisco, CA: OpenAI), Google Gemini (London, UK: Google DeepMind), Microsoft Bing (Redmond, WA: Microsoft), and Claude 3.5 (San Francisco, CA: Anthropic Inc.). Each question with answer choices was entered into an incognito window of the AI tools without any context. A chi-square test was used to assess if the percentage of correct answers varied by question difficulty and discipline, with a significance level of p<0.05. Data analysis was conducted using Stata 18.5 (StataCorp LLC: College Station, TX).

Results

Claude 3.5 achieved the highest overall accuracy with 65.7% correct responses, outperforming Google Gemini (55.3%), ChatGPT (55.0%), and Microsoft Bing (53.9%). While ChatGPT, Google Gemini, and Microsoft Bing did not show significant accuracy variations by discipline (p=0.548, p=0.145, and p=0.797, respectively), Claude 3.5 demonstrated significant performance differences across disciplines (p=0.001), mastering lower extremity (86%), dialysis access (80%), cerebrovascular (77%), venous lymph (70%), and vascular medicine (68.9%).

Conclusion

Claude 3.5 outperformed other LLMs in solving Vascular Surgery Qualifying Examination version 6 (VSQE6) questions and shows promise as a supplementary tool in vascular surgery education. LLMs are well-versed in the topics of lower extremity vascular issues, dialysis access, and cerebrovascular conditions. At this time, current LLM capabilities do not fully meet the evolving needs of vascular surgery education. While traditional methods remain essential for vascular surgery, updated models of LLMs may provide more substantial benefits in the future.

## Introduction

The invention of large language models (LLMs), such as ChatGPT, is transforming a number of fields including medicine. Applications such as ChatGPT (San Francisco, CA: OpenAI), Google Gemini (London, UK: Google DeepMind), Microsoft Bing (Redmond, WA: Microsoft), and Claude (San Francisco, CA: Anthropic Inc.) have shown promising results from question solving to support for clinical decision-making. The recent literature is full of performance-related studies using LLM for medical licensing and board exam questions, even showing mastery in highly specialized fields in medicine [1-5].

Vascular surgery is a specialty that requires years of expertise and precision. The field entails complex procedures that require an in-depth understanding of cardiovascular anatomy, physiology, and pathology with considerable expertise in a broad range of surgical skills from minimally invasive approaches to complex reconstructions. Vascular surgeons focus their medical treatment on disorders of peripheral blood vessels, outside the thoracic cavity. While the literature on surgical specialties is still growing, the utilization of LLMs in vascular surgery has never been discussed.

The Vascular Education and Self-Assessment Program (VESAP) is an online learning and self-assessment tool provided by the Society of Vascular Surgeons, consisting of more than 600 questions with detailed discussions and references. VESAP is a comprehensive, self-directed curriculum intended for vascular residents, fellows, and surgeons preparing for the Vascular Surgery Qualifying Examination (VSQE), Vascular Surgery Certification Examination (VSCE), and Continuous Certification Assessment in Vascular Surgery. Currently, there is scarce research specifically examining the effectiveness of LLMs in preparing for vascular surgery board certification.

Our study aimed to establish the relative effectiveness of the following four main LLMs: ChatGPT 3.5, Google Gemini, Microsoft Bing, and Claude 3.5. Subsequently, we utilized a curated set of VESAP version 6 multiple-choice questions to explore the possible value of AI tools as supplements to education in vascular surgery. This study investigates the utility of LLMs in vascular surgery and is one of the few to conduct a comparative analysis of LLM performance.

## Materials and methods

Study design and data collection

This study aimed to evaluate the performance of four large language models (LLMs) - ChatGPT 3.5, Google Gemini, Microsoft Bing, and Claude 3.5 - on a set of questions from the Vascular Education and Self-Assessment Program (VESAP) version 6. The dataset consisted of 642 multiple-choice questions and spanned a variety of vascular topics. Only text-based multiple-choice questions were included in the analysis, resulting in 269 questions being analyzed. This was done to maintain consistency in comparison across models. Questions that required image interpretation (e.g., clinical images or radiologic studies) were excluded from the analysis (143 questions), as some LLMs in this study did not have the capability to interpret visual data. The questions spanned key vascular surgery disciplines such as aortoiliac disease, cerebrovascular disease, dialysis access, renal and mesenteric, upper extremity, lower extremity, vascular medicine, and venous and lymphatic.

Classification of questions

Each question was classified by its question stem into one of the following three levels of cognitive complexity: (1) knowledge-based questions - questions testing recall or recognition of basic facts and concepts; (2) diagnostic questions - questions requiring interpretation of clinical scenarios to reach a diagnosis; (3) evaluation and management questions - questions testing decision-making in the management of vascular conditions, including patient evaluation and treatment strategies.

Procedure for input and data collection

To ensure standardization, one independent reviewer was tasked with copying and pasting each of the 269 questions and their corresponding answer options into the interface of each AI tool (ChatGPT 3.5, Google Gemini, Microsoft Bing, and Claude 3.5). The reviewer used incognito or private browsing windows for each LLM to avoid the influence of prior interactions, personalization, or caching of responses. No prompts or hints were provided to the AI models; the questions were entered exactly as they appeared in the VESAP6 question bank, maintaining their original structure and content to simulate the experience a human user would encounter when using the VESAP6 question bank.

Each AI tool was tasked with generating an answer for the multiple-choice question without any further input or context. The generated answers were recorded for later comparison with the correct answers as provided by VESAP6.

Statistical analysis

To compare the performance of the AI models, the generated answers from each LLM were categorized as correct or incorrect, based on the reference answer key from VESAP6. The data were further categorized by question complexity and subject matter to explore potential differences in LLM performance across these variables. A chi-square test was used to determine if there was a statistically significant difference in the percentage of correct answers based on the following factors: (a) question difficulty - knowledge-based, diagnostic, or evaluation and management; and (b) subject matter - the specific vascular discipline (e.g., aortoiliac disease, cerebrovascular disease).

A significance level of p<0.05 was set for the analysis. All data analysis was conducted using Stata version 18.5, with descriptive statistics used to summarize overall performance across the LLMs, and inferential statistics employed to assess differences in performance related to question complexity and subject matter.

Integrity of the question bank

To preserve the integrity of the VESAP6 question bank, the original questions were not altered in any way during the data entry process. The questions were input in the exact order they appeared in the VESAP6 question bank, and no edits were made to the wording or format of the questions or response options.

## Results

The Claude 3.5 model demonstrated mastery of vascular surgery content compared to other LLMs evaluated. It correctly answered 65.7% of questions (177 out of 269) in terms of the overall accuracy of the VESAP6 data set. In comparison, Google Gemini answered 148 out of 269 questions, achieving 55.3%. ChatGPT 3.5 was accurate 55.0% of the time, earning the right answer on 149 out of 269 questions. A comprehensive comparison of the accuracy scores for the four LLMs is presented in Table 1 below.

**Table 1:** Accuracy comparison of LLMs on VESAP6 questions. Overall, Claude had the highest percentage correct of all the LLMs (65.7%). This was followed by Google Gemini (55.3%), ChatGPT (55.0%), and Microsoft Bing (53.9%), respectively. LLM: large language models; VESAP6: Vascular Education and Self-Assessment Program version 6

LLM	Score (out of 169)	Percentage
ChatGPT	149/269	55.0%
Google Gemini	148/269	55.3%
Microsoft Bing	145/269	53.9%
Claude	177/269	65.7%

While ChatGPT, Google Gemini, and Microsoft Bing did not exhibit significant accuracy variations by topic (p=0.548, p=0.145, and p=0.797, respectively), Claude 3.5 showed significant performance differences across topics (p=0.001). Claude 3.5 excelled in areas such as lower extremity (9; 86.2%), dialysis access (9; 80%), cerebrovascular (27; 77%), venous and lymphatic (19; 70%), and vascular medicine (19; 68.9%). Notably, one of the most significant findings of this study was Claude 3.5’s exceptional performance in these disciplines. Results are shown in Table 1.

For evaluation and management questions, ChatGPT’s likelihood of providing correct answers decreased (p=0.010). Similarly, Google Gemini’s accuracy declined for these questions (p=0.016). In contrast, Microsoft Bing showed no significant change in accuracy with management questions (p=0.980). Claude 3.5’s accuracy also decreased as question difficulty increased (p=0.001). Notably, Microsoft Bing achieved the highest accuracy in evaluation and management questions with a correct response rate of 53.3% (p=0.980) (Table 2).

**Table 2:** Accuracy comparison of LLMs on VESAP6 questions by discipline. There was a significant difference in percentage correct by topic when using Claude (p=0.001), with Claude demonstrating superiority in five disciplines. LLM: large language models; VESAP6: Vascular Education and Self-Assessment Program version 6

Category	ChatGPT 3.5	Google Gemini	Microsoft Bing	Claude 3.5
Abdominal	66.7%	50.0%	50.0%	66.7%
Aortoiliac disease	53.6%	55.0%	50.0%	64.3%
Cerebrovascular	60.0%	62.9%	60.0%	77.1%
Dialysis access	43.3%	60.0%	70.0%	80.0%
Lower extremity	65.5%	72.4%	51.7%	86.2%
Peripheral arterial	50.0%	50.0%	50.0%	16.7%
Radiation safety	50.0%	50.0%	61.1%	64.7%
Renal and mesenteric	39.3%	32.1%	39.3%	35.7%
Upper extremity	56.7%	53.3%	53.3%	50.0%
Vascular medicine	72.4%	65.5%	48.3%	68.9%
Venous and lymphatic	55.6%	55.6%	59.3%	70.4%
Peripheral venous	16.7%	33.3%	20.0%	50.0%

## Discussion

Since the integration of artificial intelligence in education, the medical community has expressed both excitement and reasonable apprehension [5]. Undoubtedly, LLMs offer promising opportunities to enhance training by personalizing learning and knowledge acquisition. Still, however, there remains concern about our over-reliance on technology and, importantly, the potential loss of hands-on experience that is quintessential to surgery specifically [6].

The performance of LLMs, as demonstrated by our study using the VESAP6 dataset, reveals potential for AI integration in vascular surgery education [4]. Claude 3.5’s impressive accuracy of 65.7% highlights the rapid advancement of AI utility in even the most specialized and complex medical specialties [1]. Due to reporting limitations enforced by the American Board of Surgery, question-level feedback and average scores of examiners were not available for comparing LLM performance with that of human test takers.

Performance analysis of LLMs

The LLMs exhibited widely differing performances, highlighting the importance of choosing the right tool to meet the specific educational need. While Claude 3.5 came out on top and delivered a great performance in vascular surgery questions, particularly lower extremity and dialysis access, the consistency of Microsoft Bing across different question difficulty levels represents another important observation from our study. Bing was able to tackle challenging questions that require both mastery of knowledge recall as well as application and integration. Overall, our results suggest that the combination of different LLMs might serve as the best educational support for learners rather than a single AI tool.

Educational implications for vascular surgery

Claude 3.5 showed exceptional performance in several vascular surgery disciplines, which positions it as the premier LLM for vascular surgery. Claude’s ability to match and even surpass the performance of vascular surgeons in various disciplines suggests that Claude could serve as a powerful supplement for independent study, licensing exam preparation, and continuing education in vascular surgery. Of note, Claude’s performance is unique to our study and perhaps even unique to vascular surgery, as ChatGPT 4.0 is generally recognized as the most accurate LLM for solving medical board examination questions [1,4,7-10]. Based on the findings of this study, we believe LLMs can be leveraged to create interactive case-based learning platforms for vascular surgery education, offering detailed clinical scenario explanations and generating adaptive learning paths customized to each trainee’s performance, thereby improving skill acquisition and personalized learning experiences.

Current limitations and future directions

While our findings highlight the promising potential of LLMs in vascular surgery education, it is essential to acknowledge several key limitations. We recognize that the dataset used in our study, comprising fewer than 300 questions, is small and may not fully capture the scope of knowledge required in vascular surgery. In addition, the dataset was collected by a single user, which introduces the possibility of inter-user variability - a known issue when evaluating LLM performance. Responses can differ subtly or significantly depending on the phrasing and delivery of prompts, and future studies should explore the consistency of outputs across multiple users to ensure reliability and reproducibility.

Equally important to mention is the fact that LLM models are rapidly evolving. At the time of this research, we used the available LLM models that did not require a paid subscription and allowed the least number of hourly limitations, ranging from 4-8 hours, between questions. The most recent LLM versions may achieve higher accuracy rates than those observed in our study. Thus, if we were to repeat the study using the latest LLMs, we could expect improved performance.

While image-based questions were excluded in this study, we recognize that image interpretation plays an integral role in vascular surgery. Future studies should evaluate the progressively advancing capabilities of ChatGPT, including its performance with image data [11]. As LLM models advance, they could also be integrated into virtual reality simulations, offering hands-on training opportunities that closely mimic real-life surgical scenarios, further enhancing skill development and clinical decision-making in a safe, controlled environment [12].

Another limitation of this study is not using any prompts while testing the LLMs. An example of a prompt would be “You are a vascular surgeon studying for VSQE. Pick the multiple choice answer that best answers the question in the text.” However, we did not include these in our study to judge the base-level performance of the LLMs under the same conditions, thus making comparative analyses possible. We recognize that our approach and decision to input questions without context may have resulted in an underestimation of LLM’s full potential.

Lastly, our study lacked official scoring information for the VSQE. While we, like test candidates, utilized the VESAP6 question bank to simulate the VSQE, it is important to note that we did not have access to actual exam questions.

Broader implications for surgical education

These findings extend beyond vascular surgery, suggesting potential applications in other surgical specialties as well as academic surgery on the whole [13]. The ability of LLMs to process and apply vast amounts of medical knowledge could revolutionize how surgical education is approached, offering personalized learning experiences and on-demand access to expert-level information.

While the integration of AI in surgical education presents exciting opportunities, it is important to approach this integration thoughtfully [14]. LLMs should be viewed as complementary tools to enhance, rather than replace, traditional teaching methods and hands-on clinical experience. As these technologies continue to evolve, ongoing evaluation of their performance, limitations, and impact on learning outcomes will be crucial in shaping the future of surgical education [15,16]. LLMs may support the creation of automated, evidence-based curricula that continuously evolve with new research and guidelines.

## Conclusions

Claude 3.5 emerged as the best-performing LLM for vascular surgery, achieving an accuracy of 65.7% on the VESAP6 dataset, outperforming Google Gemini and ChatGPT 3.5. Its strong performance in lower extremity and dialysis access questions highlights its potential as a valuable tool for vascular surgery education. Although Microsoft Bing had the highest accuracy on the most difficult questions, Claude 3.5’s overall performance makes it a standout option. These findings suggest that while no single LLM excels in all areas, Claude 3.5 and Bing offer promising applications in vascular surgery education.
